# *In Silico* Analysis of Putative Sugar Transporter Genes in *Aspergillus niger* Using Phylogeny and Comparative Transcriptomics

**DOI:** 10.3389/fmicb.2018.01045

**Published:** 2018-05-18

**Authors:** Mao Peng, Maria V. Aguilar-Pontes, Ronald P. de Vries, Miia R. Mäkelä

**Affiliations:** ^1^Fungal Physiology, Westerdijk Fungal Biodiversity Institute and Fungal Molecular Physiology, Utrecht University, Utrecht, Netherlands; ^2^Department of Microbiology, Faculty of Agriculture and Forestry, University of Helsinki, Helsinki, Finland

**Keywords:** *Aspergillus niger*, sugar transporter, phylogeny, comparative transcriptomics, fungi

## Abstract

*Aspergillus niger* is one of the most widely used fungi to study the conversion of the lignocellulosic feedstocks into fermentable sugars. Understanding the sugar uptake system of *A. niger* is essential to improve the efficiency of the process of fungal plant biomass degradation. In this study, we report a comprehensive characterization of the sugar transportome of *A. niger* by combining phylogenetic and comparative transcriptomic analyses. We identified 86 putative sugar transporter (ST) genes based on a conserved protein domain search. All these candidates were then classified into nine subfamilies and their functional motifs and possible sugar-specificity were annotated according to phylogenetic analysis and literature mining. Furthermore, we comparatively analyzed the ST gene expression on a large set of fungal growth conditions including mono-, di- and polysaccharides, and mutants of transcriptional regulators. This revealed that transporter genes from the same phylogenetic clade displayed very diverse expression patterns and were regulated by different transcriptional factors. The genome-wide study of STs of *A. niger* provides new insights into the mechanisms underlying an extremely flexible metabolism and high nutritional versatility of *A. niger* and will facilitate further biochemical characterization and industrial applications of these candidate STs.

## Introduction

*Aspergillus niger* is a filamentous ascomycete fungus, which is found in a wide range of biotopes on earth and has a long history of use for the industrial production of hydrolytic enzymes (Culleton et al., 2013) and organic acids (Andersen et al., 2011). It can efficiently degrade all major polysaccharide components of the plant cell wall (cellulose, hemicellulose, and pectin) by secreting a versatile set of carbohydrate active enzymes (CAZymes) (de Vries and Visser, 2001). During the last decades, the extracellular enzymes and associated transcriptional factors (TFs) involved in fungal plant biomass degradation have been extensively studied (de Vries and Visser, 2001; Huberman et al., 2016; Benocci et al., 2017). However, the sugar transporters (STs) that are essential for taking up the mono- and short oligosaccharides, resulting from extracellular enzymatic digestion of lignocellulose, into the fungal cell have not been systematically investigated. Compared to the nearly 100 ST genes predicted in the *A. niger* genome (de Vries et al., 2017), only 10 transporters have been biochemically characterized in *A. niger* for their sugar specificity, resulting in five D-glucose transporters (Vankuyk et al., 2004; Jorgensen et al., 2007; Sloothaak et al., 2015), three D-xylose transporters (Sloothaak et al., 2016b), one D-galacturonic acid transporter (Sloothaak et al., 2014), and one L-rhamnose transporter (Sloothaak et al., 2016a).

Sugar transporters are ubiquitously present in all kingdoms of life from bacteria to fungi, plants, and animals. Most STs belong to the sugar porter family (Pfam ID: PF00083), which is a subfamily of the major facilitator superfamily (Pfam ID: PF07690). The ST protein typically contains 12 transmembrane helices (TMH) (Abramson et al., 2003) and several well-defined ST signatures (Joost and Thorens, 2001). Previous studies of STs in the model organism, yeast *Saccharomyces cerevisiae*, and recent comparative genomic investigations have shed light on the genetic variation and evolutionary adaptation of fungal STs. Firstly, the STs show clear functional redundancy in fungal genomes. This redundancy is not just reflected by multiple genes encoding similar transporters, but is also evident from individual transporters with the ability to transport several different sugars (Vankuyk et al., 2004). A previous study has shown that in total 20 STs had to be deleted to completely block the hexose uptake in *S. cerevisiae* (Wieczorke et al., 1999). Secondly, the different numbers of ST genes present in fungal genomes were found to correlate with the life style of the fungus. The expansion of ST genes in genomes of the Pezizomycotina compared to the Saccharomycotina was proposed to be associated with the different approaches for carbon source utilization in nature between these two fungal classes (Cornell et al., 2007). In addition, recent 3D structure and genetic mutation experiments have revealed that the mutation of only a limited number of key amino acids could significantly change the specificity and affinity of STs (Quistgaard et al., 2013; Madej et al., 2014; Young et al., 2014).

Compared to the well-studied sugar “transportome” of *S. cerevisiae*, the corresponding knowledge for other fungi is far from complete. Besides the urgent need for functional characterization of more STs, another big challenge is the identification of the regulatory mechanisms driving ST gene expression during fungal sugar utilization. Only a few regulators, such as CreA and XlnR, have been shown to regulate expression of ST genes in *A. niger* (Vankuyk et al., 2004; Andersen et al., 2008). In addition, it is still debatable whether similar mechanisms of the well-studied yeast transceptor genes [e.g., Snf3 and Rgt2 (Horak, 2013)], which function both as ST and receptor for signal transduction, are commonly present in other fungal species (Lin and Li, 2011; Znameroski et al., 2014).

A genome-wide study of the sugar “transportome” in *A. niger* not only provides new insights on the physiological role of STs on fungal growth, but also provides new target genes for rational engineering of industrial fungal species. In this study, we first phylogenetically classified all predicted STs in the *A. niger* genome, which revealed nine different families with different putative sugar specificity and sequence features. Then we compared gene expression profiles of ST genes on different carbon sources, as well as with mutants of transcriptional regulators related to plant polysaccharide degradation, which revealed complex and dynamic expression patterns of the sugar transportome of this fungus.

## Materials and Methods

### Fungal Strains, Transcription Factor Mutants, and Cultivation

The *A. niger* strains used in this study are listed in **Table 1**. Strains were grown at 30°C on complete medium (CM) (de Vries et al., 2004) with 1.5% agar to generate spore plates. Liquid cultures were incubated on a rotary shaker at 250 rpm. Pre-cultures for RNA isolation were incubated for 16 h in 1 L Erlenmeyer flasks that contained 250 mL CM supplemented with 2% D-fructose. Mycelium was washed with minimal medium (MM) (de Vries et al., 2004) and 1 g (wet weight) aliquots were transferred for 2 h to 250 mL Erlenmeyer flasks containing 50 mL MM supplemented with 25 mM mono- or disaccharide, or 1% polysaccharide (Gruben et al., 2017). The only exceptions were D-maltose cultures of N402 and Δ*amyR* strains that were incubated for 4 h and for which 1% maltose was used as a carbon source (vanKuyk et al., 2012). All carbon sources are listed in Supplementary File 4. Mycelium was harvested by vacuum filtration, dried between towels and frozen in liquid nitrogen. All cultures were performed as biological duplicates.

**Table 1:** *Aspergillus niger* strains used in this study.

Strain	Genotype	Reference
N402	*cspA1*	Bos et al., 1988
FP-304 (Δ*rhaR*)	*cspA1*, Δ*kusA*::*amdS*+, *pyrA5*, Δ*rhaR*::*pyrA*+	Gruben et al., 2014
UU-A101.1 (Δ*amyR*)	*cspA1*, Δ*argB, pyrA6, leuA1, nicA1*, Δ*amyR*::*argB*+	vanKuyk et al., 2012
FP-306 (Δ*galX)*	*cspA1*, Δ*kusA*::*amdS*+, *pyrA5*, Δ*galX*::*pyrA*+	Gruben et al., 2012
UU-A033.21 (Δ*araR)*	*cspA1, pyrA6, nicA1, leuA1*, Δ*argB*::pIM2101 Δ*araR*::*argB*+	Battaglia et al., 2014
UU-A062.10 (Δ*xlnR)*	*cspA1, pyrA6*, Δ*argB, nicA1, leuA, pyrA6*, Δ*xlnR*::*pyrA*+	Battaglia et al., 2014
JN35.1	*cspA1, kusA*::*DR-amdS-DR, gaaR*::*hygB*	Alazi et al., 2016

### Identification of Sugar Transporters

The proteome of *A. niger* CBS 513.88 was downloaded from the AspGD database^1^. A total of 61 fungal ST protein sequences were collected from a manual literature search. The ST domain (PF00083) profile extracted from the PFAM database^2^ was used to search against the combined sequence files of the *A. niger* proteome and known transporters with the “hmmsearch” of the HMMER tool (Eddy, 1998). The hmmsearch score ≥238 was chosen as a cutoff to define the ST candidates, since it was the lowest score observed among the results of all the known transporters.

### Phylogenetic Analysis

All the collected ST sequences were aligned using the transmembrane protein alignment tool, TM-Coffee software (Floden et al., 2016), with default parameters. Positions with too many gaps (>20%) were excluded from the alignment. Subsequently, RAxML (Stamatakis, 2014) was used for phylogenetic analysis with 500 bootstraps and PROTGAMMAWAG option. STs from *Arabidopsis thaliana* (Buttner, 2010) were used as an outgroup in the phylogenetic analysis. The resulting gene tree was visualized using iTOL (Letunic and Bork, 2016). The conservation of well-described sugar motifs was checked on the alignment sequences. Sequence logos were generated with the online tool WebLogo^3^ (Crooks et al., 2004).

### Transcriptome Analysis

Microarrays were used to evaluate the genome-wide gene expression in the wild type strain grown on different carbon sources and regulatory mutants grown on their specific inducing compounds. RNA was extracted using the TRIzol reagent (Invitrogen) and purified using TRIzol^®^ Plus RNA Purification Kit (Sigma-Aldrich) according to the instructions of the manufacturer. The RNA concentration was calculated from the absorbance at 260 nm in a spectrophotometer (Biochrom Libra S22). The RNA quality was analyzed with an Agilent 2100 Bioanalyzer using a RNA6000 LabChip kit (Agilent Technologies). Microarray hybridization was performed at GenomeScan (Leiden, Netherlands). All the raw microarray data, which has recently been used for expression-based clustering of CAZymes (Gruben et al., 2017), is deposited in the GEO database with Accession No. GSE98572.

The microarray raw data was normalized and summarized using the robust multi-array analysis algorithm (RMA) (Irizarry et al., 2003). The average value of each gene’s normalized expression was calculated for fungal samples from the same carbon source or regulatory mutant. These gene expression values were visualized with heatmap using R package “gplots.”

To compare the wild type and TF mutant strains, the Limma package (Ritchie et al., 2015) of R was used to discover the significantly expressed genes. Fold change of 1.5 and adjusted *p*-value of 0.01 were used as cutoffs. The RNA-seq data for GaaR was extracted from a previous publication (Alazi et al., 2016) and the original threshold (FPKM values >10, fold change >1.5, and *t*-test *p*-value < 0.05) was used to select the significantly expressed genes.

## Results

### Phylogenetic Analysis of *A. niger* Sugar Transporters

**FIGURE 1:**
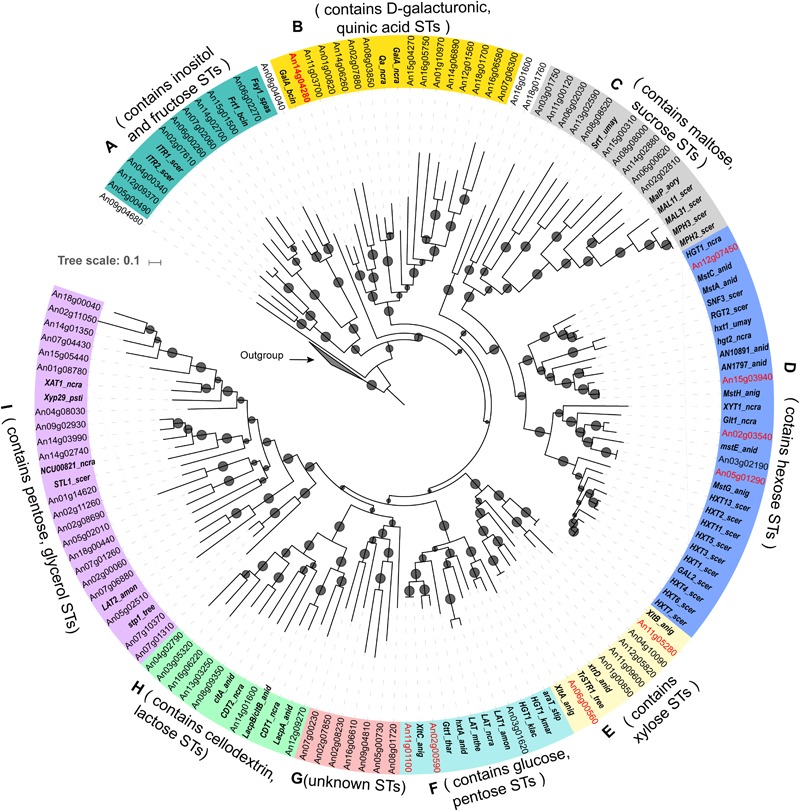
Phylogenetic classification of (putative) sugar transporters in *Aspergillus niger*. The tree contains 86 sugar transporters of *A. niger* containing Pfam domain PF00083 and 61 experimentally characterized fungal sugar transporters. The gene names of previously experimental characterized sugar transporters of *A. niger* are highlighted with red fonts and the literature reported transporters from other fungi species are highlighted with bold italic fonts. Potential substrates for members of each clade are suggested based on the functions of the known transporters included in the specific clades. *Arabidopsis thaliana* sugar transporters (Buttner, 2010) were used as an outgroup. Branches with bootstrap values >60% were indicated with small circles. The abbreviation of fungal species name is attached to each known transporter gene (anid = *Aspergillus nidulans*, anig = *Aspergillus niger*, aory = *Aspergillus oryzae*, amon = *Ambrosiozyma monospora*, bcin = *Botrytis cinerea*, kmar = *Kluyveromyces marxianus*, klac = *Kluyveromyces lactis*, ncra = *Neurospora crassa*, psti = *Pichia stipitis*, scer = *Saccharomyces cerevisiae*, spas = *Saccharomyces pastorianus*, stip = *Scheffersomyces stipitis*, tree = *Trichoderma reesei*, thar = *Trichoderma harzianum*, umay = *Ustilago maydis*).

By searching the conserved ST domain (Pfam ID: PF00083) in the proteome of *A. niger*, a total of 86 putative ST genes were identified. The protein sequences of all predicted *A. niger* ST genes together with 61 literature reported STs in other fungi were used for phylogenetic analysis (Supplementary File 1). Eighty-three of the 86 predicted SP proteins of *A. niger* fell into nine different clades supported by bootstrap values above 60%, while three of predicted STs were not located in the main clades (**Figure 1**).

Based on the previously reported biochemical properties of the 61 characterized STs included in the phylogenetic tree, we could assign putative sugar specificity to each clade. The result is consistent with the recently published phylogenetic study of transporters from eight *Aspergillus* genomes (de Vries et al., 2017). Clade A contains nine *A. niger* STs and two yeast myoinositol transporters ITR1 and ITR2 (Nikawa et al., 1991), as well as one fructose transporter from *Botrytis cinerea* (Frt1) (Doehlemann et al., 2005), and one fructose porter from *Saccharomyces pastorianus* Fsy1 (Galeote et al., 2010). Clade B contains the D-galacturonic acid transporters from *A. niger* (GatA) (Sloothaak et al., 2014) and *B. cinerea* (Zhang et al., 2014), and one quinic acid transporter (Tang et al., 2011). Clade C comprises 10 *A. niger* STs, four maltose transporters from yeasts (MAL11, MAL31, MPH2, and MPH3) (Horak, 2013), one maltose permease from *Aspergillus oryzae* (Hasegawa et al., 2010) and one sucrose transporter from *Ustilago maydis* (Srt1) (Wahl et al., 2010). Clade D contains five *A. niger* STs, several well-characterized yeast hexose transporters (Ozcan and Johnston, 1999), two glucose sensors [SNF3 and RGT2 (Ozcan and Johnston, 1999)], as well as known D-glucose transporters from *Neurospora crassa* [Hgt1 and Glt1 (Wang B. et al., 2017)], and other *Aspergillus* species [*mstA, mstC*, and *mstE* (Forment et al., 2006) of *A. nidulans*]. Clade E contains recently characterized D-xylose transporters from *A. niger* ATCC 1015 and *Trichoderma reesei* (Sloothaak et al., 2016b). Clade F contains diverse pentose and hexose transporters including L-arabinose [LAT1 (Verho et al., 2011) and AraT (Subtil and Boles, 2011)], D-xylose [AnXltC (Sloothaak et al., 2016b)], and D-glucose [*gtt1* (Delgado-Jarana et al., 2003)] transporters. No known transporter is present in Clade G. Clade H includes lactose permeases from *A. nidulans* [*lacpA* and *lacpB* (Fekete et al., 2012, 2016)] and cellodextrin transporters from *N. crassa* [CDT1 and CDT2 (Znameroski et al., 2014)]. Clade I mainly contains pentose transporters [XAT1 (Li et al., 2014), LAT2 (Verho et al., 2011), NCU00821 and Xyp29 (Du et al., 2010)] and a yeast glycerol transporter [Stl1 (Ferreira et al., 2005)].

### Protein Structural Features of Phylogenetic Clades

**FIGURE 2:**
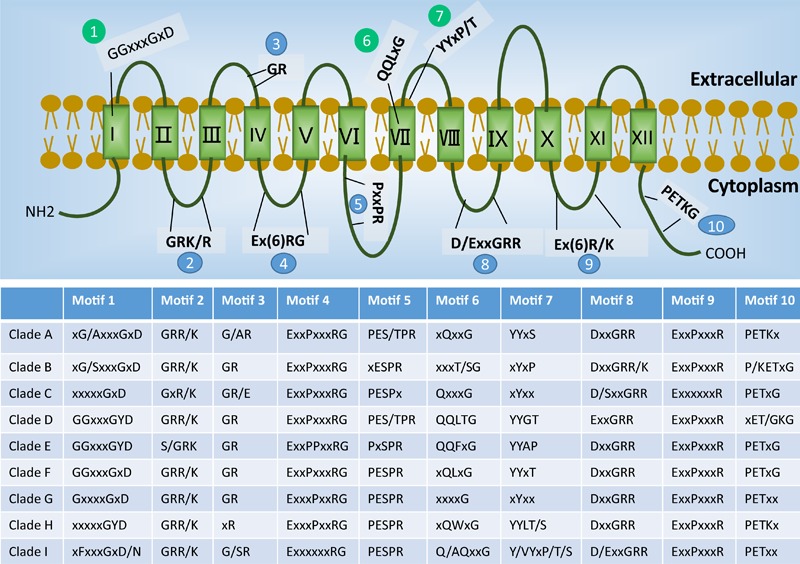
Sugar transporter signatures present in each phylogenetic clade (see **Figure 1**). The overall distribution of well-documented motifs is shown on the top of the schematic figure, where blue color indicates the conserved structure signatures and green color indicates the known sugar binding motifs. The table at the bottom shows the conservation and variations of the motifs found in each clade, where the “/” indicates an alternative amino acid and “x” indicates a non-specific amino acid.

To examine the diversity of each different phylogenetic clade of STs, the well-documented structural features of STs were checked for their presence in the sequence alignment (**Figure 2** and Supplementary Files 2, 3). Overall, the protein sequences of the 86 STs share 14–73% sequence identity. The N- and C-terminal parts showed the most diversity. Common to all putative STs are the predicted 12 TMHs (Abramson et al., 2003). In addition, the STs contain almost all the well-defined ST signatures (Joost and Thorens, 2001) (e.g., the motifs 2, 3, 4, 5, 8, 9, and 10 shown in **Figure 2**). The broad conservation of these motifs across different ST clades indicates their critical roles in the structure and function of STs. The exceptions include the glycine of motif 3, the first proline of motif 5, and the proline of motif 10, which are not conserved in Clades H, B, and D, respectively.

In contrast to the conserved ST signatures (Joost and Thorens, 2001), striking differences were observed for key motifs that were reported to determine the sugar specificity. These motifs include the GGxxxGxD motif in the first TMH region (Young et al., 2014; Wang C. et al., 2017), and QQLxG motif (Seatter et al., 1998; Sun et al., 2012) and YYxP/T motif (Wang et al., 2016) in the seventh TMH region (shown, respectively, as motifs 1, 6, and 7 in **Figure 2**). The triple glycine motif GGxxxGxD was predominately found in Clades D, E, and F, which comprise the common hexose and pentose transporters. The previously identified glucose binding site QQLxG was conserved in all members of Clade D (hexose transporters), while large variation is present in other clades. Similarly, the YYxP/T motif, which was reported to determine glucose and xylose specificity (Wang et al., 2016), was absent in the non-pentose/hexose Clades B, C, and G.

### Expression Profiles of Sugar Transporter Genes on Different Carbon Sources

**FIGURE 3:**
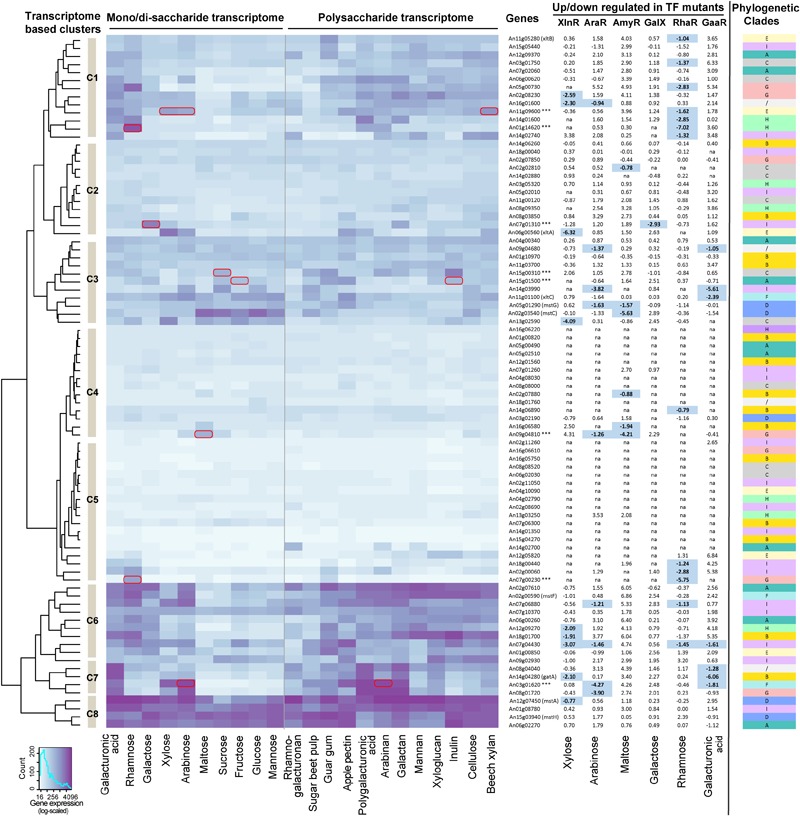
Expression profiles of sugar transporter encoding genes in *A. niger* wild type and transcriptional factor (TF) mutant strains during growth on diverse carbon sources. The color from light to dark indicates a gene expression level from low to high. Hierarchical clustering of gene expression is shown on the left side of the heatmap. Genes with specific sugar induced expression pattern are marked with a red box on the heatmap. The table of log2-based fold change between the *A. niger* wild type and TF mutants is shown on the right side of the heatmap. The carbon source used in each transcriptome is indicated at the bottom of the heatmap. Expression values <50 in both wild type and regulatory mutant strains were ignored for calculating fold change and indicated with “na” in the figure. Genes with fold change >1.5 and *p*-value < 0.01 in the microarray datasets were considered as proof of regulator function and are depicted in bold and highlighted in pale blue [the only exception is the GaaR RNA-seq datasets, where the original threshold (Alazi et al., 2016) was applied]. The phylogenetic clade of each ST is shown on the right side of the figure.

Based on the diverse phylogenetic and structural features across different ST subfamilies, we were curious whether different ST families may harbor different expression patterns during growth on different carbon sources. To investigate this, we compared microarray-based transcriptomes of *A. niger* grown on 10 mono- or disaccharides and 12 polysaccharides (**Figure 3** and Supplementary File 4). Several interesting expression patterns of transporters were observed. Firstly, the transporters from the same phylogenetic clade did not share similar expression profiles (**Figure 3**). Secondly, several transporters from Clade A (An06g02270), Clade D (An12g07450/*mstA* and An15g03940/*mstH*), and Clade I (An01g08780) were well-expressed in almost all the tested carbon sources (Cluster C8 in the heatmap of **Figure 3**), which supports their broad sugar specificity and essential role for fungal physiology. A clear example is the high-affinity glucose transporter encoded by *mstA*, which was shown to be able to transport D-glucose, D-mannose, and D-xylose, and to be important for fungal growth (Vankuyk et al., 2004). In contrast, some transporters (An08g08520, An16g05750, An16g06610, An15g04270, and An14g01350 in Cluster C5 of **Figure 3**) were lowly expressed in most of the transcriptomes, which indicates a less important function under the tested conditions and sampling times.

In addition, several transporter encoding genes showed clear sugar specific expression patterns on the tested carbon sources. For instance, the low-affinity D-glucose transporter encoding gene *mstC* (An02g03540) and high-affinity D-glucose transporter encoding gene *mstG* (An05g01290) were higher expressed on hexose substrates than other monosaccharides. The D-galacturonic acid transporter encoding genes *gatA* (An14g04280) (Sloothaak et al., 2014) and the D-xylose transporter encoding gene *xltA* (An06g00560) (Sloothaak et al., 2016b) were also clearly induced by their corresponding sugars. Interestingly, several transporters lacking solid experimental proof of function also had highly specific expression profiles. For example, the gene An15g01500 was significantly up-regulated on D-fructose, sucrose, pectin, and inulin, indicating that it may be a D-fructose transporter. Two potential disaccharide transporters from Clade C, An15g00310 and An09g04810, showed induced expression patterns on sucrose and maltose, respectively. Consistent with our findings, the An15g01500 and An09g04810 genes were shown to be able to complement the *S. cerevisiae* transport mutant strains grown on D-fructose and maltose medium, respectively (de Vries et al., 2017). Two putative pentose transporters, An11g09600 and An03g01620, were highly expressed on D-xylose and L-arabinose. Furthermore, two transporters encoding gene specifically induced by L-rhamnose (An07g00230 and An01g14620) and one induced by a D-galactose (An07g01310) were detected.

### Transcriptional Regulation of Sugar Transporters

The dynamic expression patterns of different STs suggest that their expression is well-controlled by a sophisticated transcriptional regulatory system. In line with this hypothesis, several important fungal TFs involved in degradation of plant biomass polysaccharides, such as XlnR (de Souza et al., 2013) and Clr1/Clr2 (Craig et al., 2015), have been reported to regulate the expression of ST genes in addition to affecting the process of plant biomass degradation. Here we compared the gene expression of the transporters in TF mutants of XlnR, AraR, AmyR, GalX, RhaR, and GaaR with the wild type *A. niger* strain during growth on D-xylose, L-arabinose, maltose, D-galactose, L-rhamnose, and D-galacturonic acid, respectively (**Figure 3** and Supplementary File 4). The significantly reduced gene expression profiles (fold change >1.5 and *p*-value < 0.01) showed that XlnR affects the expression of a broad range of ST encoding genes, such as *mstA, gatA, xltA*, and six putative transporters. AraR affects the expression of nine putative transporter encoding genes, two of which are also regulated by XlnR (An16g01600 and An07g04430). There are six transporter encoding genes that were regulated by AmyR, mainly from Clades B, C, and D, which contain hexose, maltose, sucrose, and D-galacturonic acid transporters. Only one transporter encoding gene was regulated by GalX, which was specifically induced by D-galactose (An07g01310). The RhaR mutant affected in total 13 transporters, seven of which were from Clades I and E, which contains mainly pentose transporters. In the GaaR mutant, seven transporter encoding genes had reduced expression, including *gatA* and *xltC*.

Interestingly, nine transporter encoding genes were affected by more than one TFs. One putative transporter (An07g04430) was affected by four TFs (XlnR, AraR, RhaR, and GaaR). This gene was highly expressed on a broad range of polysaccharides indicating that it may function as an important transporter during plant biomass degradation. In addition, there are two transporters affected by both XlnR and AraR, two transporters affected by AraR and AmyR, two transporters affected by AraR and RhaR, one transporter affected by XlnR and RhaR, one transporter affected by GaaR and RhaR, and four transporters affected by AraR and GaaR (**Figure 3**). This last result may be due to the previously suggested co-regulatory effect of AraR on GaaR (Kowalczyk et al., 2017).

## Discussion

The phylogenetic analysis identified nine major families consisting of 86 *A. niger* (putative) STs with specificity to different groups of sugar molecules including hexoses, pentoses, di-/oligosaccharides, and galacturonic/quinic acid. Transcriptomes of the wild type *A. niger* grown on a broad range of carbon sources and TF mutants grown on their inducing compounds revealed that transporter genes from the same phylogenetic clade displayed very different expression patterns. Most clades contain genes that were induced by a broad range of sugars, as well as genes only induced by specific sugars. The diverse expression profiles within the phylogenetic clades could indicate that these are transporters with similar specificity, but different affinity, which would explain the difference in gene expression. For example, genes encoding a low affinity and high affinity glucose transporter would not be expected to be highly expressed at the same time as their gene products would work optimally under very different physiological conditions. Previous studies in *S. cerevisiae* have shown that it contains a large family of hexose transporters with high sequence similarity, but with different affinity (Kruckeberg, 1996; Boles and Hollenberg, 1997). These STs have been found to be distinctly expressed during different stages of wine fermentation process (Perez et al., 2005). Similarly, in our study gene expression of two different affinity hexose transporters from the phylogenetic Clade D, low affinity *mstC* and high affinity *mstF*, showed clear anti-correlation pattern between hexoses and other sugars (**Figure 3**). The *mstF* gene was highly expressed on various carbon sources, except hexoses, and hexose disaccharides, while in contrast, *mstC* was only induced on hexoses and hexose disaccharides. The multiple ST genes in each specific ST subfamily together with their dynamic expression and diverse sugar binding affinity could enable the organism to efficiently respond to a changing composition and concentration of carbon sources and contribute to its successful adaption to an extremely wide range of environments.

The comparison of STs expression of five different TF mutants suggested that genes from the same phylogenetic clade could be regulated by different transcriptional regulators, thus supporting the differential expression patterns observed within the phylogenetic clades. ST genes from the same phylogenetic clade share high sequence similarity in the gene coding region, which contribute to their similar sugar transporting specificity. However, the variability in the presence/absence of regulatory sequences in their promoter regions could enable binding of different TFs and therefore diverse expression patterns on different growth conditions. Similar transcriptional rewiring has been observed previously when GAL genes and their regulatory binding sites have been compared between *S. cerevisiae* and *C. albicans* (Dalal et al., 2016). Further evidence of the range of target genes of the various TFs and analysis of promoter sequences of ST genes in *A. niger* are required to confirm this hypothesis.

Interestingly, most of the *S. cerevisiae* hexose transporters appear together as a separate cluster in our phylogenetic tree (**Figure 1**), which is in line with previous hypothesis that the ST family originated from gene duplication specific for this (group of) species (Reifenberger et al., 1995; Leandro et al., 2009; Lin and Li, 2011). An exception is the two glucose sensors Rgt2 and Snf3 (Gancedo, 2008), which cluster together with *A. niger mstH* (An15g03940). This gene is a homolog of *A. nidulans* HxtB (Dos Reis et al., 2013), which has been suggested to be a low affinity glucose transporter that is involved in glucose signaling. Deletion of the corresponding gene results in a hyperconidiation phenotype under certain growth conditions (Dos Reis et al., 2017). Surprisingly, the *A. niger mstH* was characterized as a high affinity glucose transporter (Sloothaak et al., 2015), and is therefore unlikely to be involved in glucose sensing. *A. niger* MstH and *A. nidulans* HxtB represent an example of transporters that have high sequence homology, but show different biochemical properties. A more extensive phylogenetical and biochemical characterization of STs across the fungal kingdom would probably provide more insight into the evolution of the transceptor genes, which have function both as ST and receptor for signal transduction (Lin and Li, 2011; Znameroski et al., 2014).

During plant biomass degradation, fungi secrete extracellular enzymes to decompose the polysaccharides to small molecules, which are then imported into cell through STs and used for fungal growth and metabolism. The increasing amount of functional genomics data helps to build a more comprehensive network connecting STs, regulators, CAZymes and metabolic genes to facilitate better understanding of efficient fungal sugar utilization (Peng et al., 2017; Samal et al., 2017). In this study, we identified several transporters that are controlled by plant polysaccharide degradation related transcriptional regulators (**Figure 3**). For some of the regulators, our results confirm those of a previous study (de Souza et al., 2013), in which An03g01620 and An08g01720 were also affected by AraR, while An06g00560 (*xltA*) was under control of XlnR. While some of the transporters appear to be under control of the same regulators (e.g., XlnR, AmyR, and RhaR) that control polysaccharide degradation and sugar catabolism, interestingly, many of them have expression profiles that do not correlate with polysaccharide degradation or sugar catabolism (Gruben et al., 2017). However, more functional data on individual transporters is needed to place them accurately in the overall network of fungal plant biomass degradation.

## Conclusion

Considering that only 10 STs of *A. niger* have been functionally characterized so far, the combined phylogenetic classification and comparative transcriptome analysis in this study provides an important reference for future biochemical characterization of new ST candidates. In addition, the genome-wide investigation of the *A. niger* sugar transportome presented here is not only important in understanding the physiological role of STs for fungal growth, but also provides new target genes for rational engineering of industrial fungal species and facilitates their biotechnological applications. The low level of correlation between phylogeny and expression profiles indicates fast functional divergence as well as fast gene-regulatory evolution of STs, showing a clear need for extensive and detailed studies of these highly important proteins.

## Author Contributions

MP and RV experimental design, data analysis, and manuscript writing. MVA-P microarray data analysis. MM and RV critical revision. All authors read and approved the final manuscript.

## Conflict of Interest Statement

The authors declare that the research was conducted in the absence of any commercial or financial relationships that could be construed as a potential conflict of interest.

## Footnotes

1 http://www.aspgd.org/

2 http://pfam.xfam.org/family/pf00083

3 https://weblogo.berkeley.edu/
