# Supplementary material for: In Silico Analysis of Putative Sugar Transporter Genes in Aspergillus niger Using Phylogeny and Comparative Transcriptomics
Source: Front Microbiol. 2018 May 18;9:1045. doi: 10.3389/fmicb.2018.01045 (PMC5968117; doi:10.3389/fmicb.2018.01045)

Additional File 2: Protein sequence alignment of sugar transporters of *Aspergillus niger* where the alignment positions with gap >20% was excluded and the important motifs were highlighted.

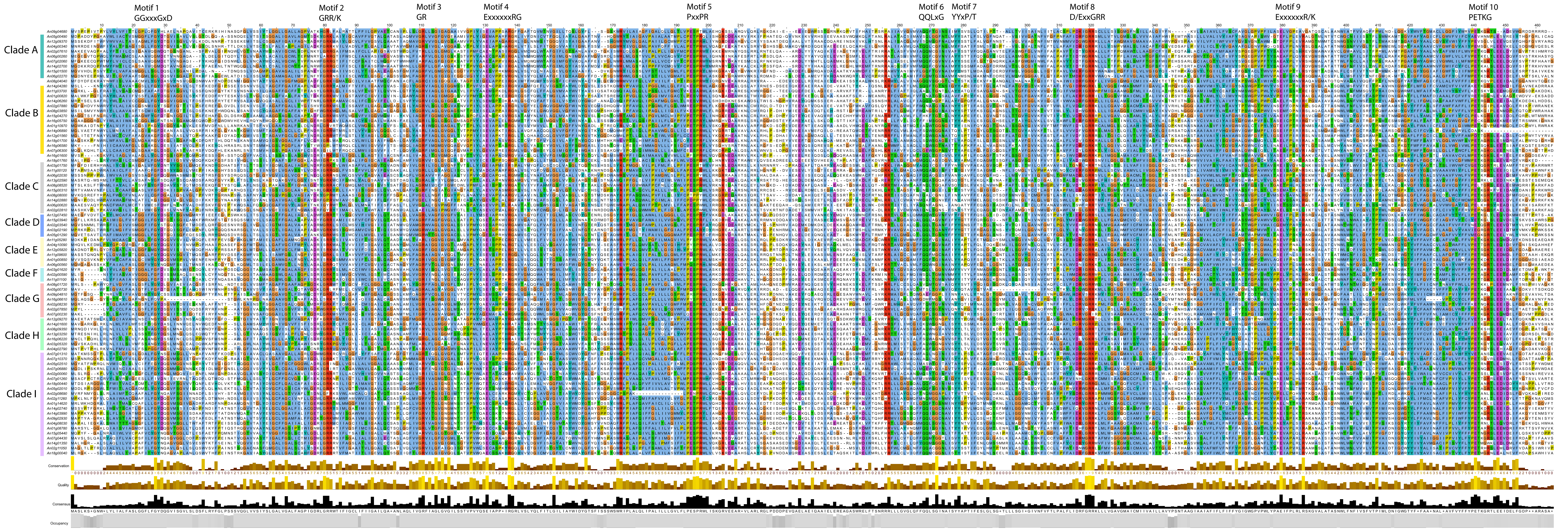

Supplement: Supplementary file 2 [file Data_Sheet_2.pdf]
