# Supplementary material for: In Silico Analysis of Putative Sugar Transporter Genes in Aspergillus niger Using Phylogeny and Comparative Transcriptomics
Source: Front Microbiol. 2018 May 18;9:1045. doi: 10.3389/fmicb.2018.01045 (PMC5968117; doi:10.3389/fmicb.2018.01045)

Additional File 3: Sequence logos for each sugar transporter family and important motifs are highlighted above.

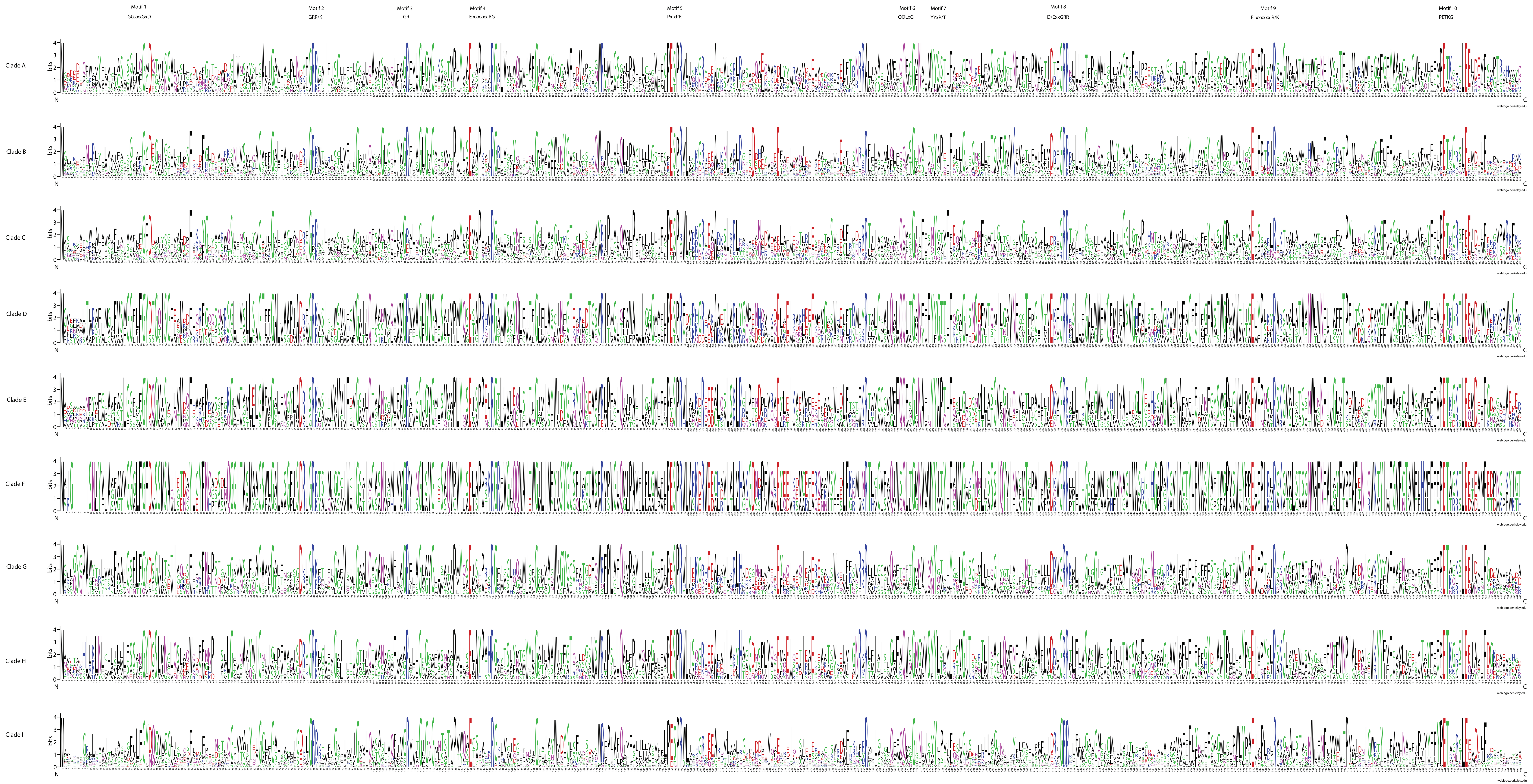

Supplement: Supplementary file 3 [file Data_Sheet_3.pdf]
